# Supplementary material for: Differing Membrane Interactions of Two Highly Similar Drug-Metabolizing Cytochrome P450 Isoforms: CYP 2C9 and CYP 2C19
Source: Int J Mol Sci. 2019 Sep 4;20(18):4328. doi: 10.3390/ijms20184328 (PMC6770661; doi:10.3390/ijms20184328)
Supplement: Supplementary file 1 [file ijms-20-04328-s001.zip › ijms_supplementary_wade.pdf]

Supporting Information for:

Differing membrane interactions of two highly similar drug metabolizing cytochrome P450 isoforms: CYP 2C9 and CYP 2C19.

Ghulam Mustafa[1,2], Prajwal P. Nandekar[1,2], Neil J. Bruce[1], Rebecca C. Wade[1,2,3*]

[1]Molecular and Cellular Modeling Group, Heidelberg Institute for Theoretical Studies (HITS), Schloß-Wolfsbrunnenweg 35, 69118 Heidelberg, Germany;

[2]Zentrum für Molekulare Biologie der Universität Heidelberg, DKFZ-ZMBH Alliance, INF 282, 69120 Heidelberg, Germany;

[3]Interdisciplinary Center for Scientific Computing (IWR), Heidelberg University, INF 368, 69120 Heidelberg, Germany

Contents:

Supporting Tables S1-S3…………………………………………………....2

Supporting Figures S1-S7………………………………………………. 6

PDB files of final frames from AA MD simulations in CYP_2C9_2C19_pdb folder

Supporting Tables

Table S1. Coarse-grained simulations carried out for CYP 2C9 and CYP 2C19.

CYP System	PDB ID	Residue numbers and regions: TM Helix	Linker	Flexible linker*	Globular domain	No. of CG simulations
		**CYP 2C9**				
S1	1R9O	3-21	22-49	22-36	50-490	10
S2	1R9O	3-21	22-49	26-38	50-490	5
S3	1R9O	**Globular domain only**				1
M1	Model1	3-21	22-49	22-36	50-490	6
M2	Model2	3-21	22-49	22-36	50-490	6
M3	Model3	3-21	22-49	22-36	50-490	6
M4	Model4	3-21	22-49	22-36	50-490	5
mtCYP2C9	Mutant#	3-21	22-49	22-36	50-490	5
		**CYP 2C19**				
S1	4GQS	3-24	25-49	26-38	50-490	10
S2	4GQS	3-21	22-49	22-36	50-490	5
S3	4GQS	**Globular domain only**				1
mtCYP2C19	Mutant	3-21	22-49	22-36	50-490	5

S: CG simulations using globular domain from crystal structure, M: CG simulations of CYP 2C9 using modeled structures, *Linker region which was set flexible by removing elastic restraints from the residues mentioned. #Mutant structures of CYP 2C9 and CYP 2C19 prepared after substitution of residues in the linker, β-strand1, B-C and F’-G’ loops.

Table S2: CG simulations of CYP 2C9 models show the dependence on the initial conformation of the globular domain of the final orientation of the globular domain with respect to the membrane. Values of angles and distances characterizing the positioning of the globular domain are shown for CG trajectories of CYP 2C9 M1 and M3, along with their classification according to the orientation of the globular domain into classes A, A/B or B. The results are shown for trajectories starting from six different initial conformations of the full protein with different orientations of the globular domain (Ori1-6). Values of means and standard deviations were computed after excluding the first 5 μs of each production simulation.

**CYP 2C9 initial orientations**	**Angles (°)**		**Distances (Å)**			**Class**
	α	β	Linker	F'-G'	Globular	
			**CYP2C9:M1**			
**Ori1**	88±6	110±6	20±2	25±2	46±2	A
**Ori2***	90±7	102±12	19±2	24±2	46±2	A
**Ori3**	101±6	123±6	21±2	26±2	47±2	A
**Ori4**	99±7	123±7	20±2	25±2	46±2	A
**Ori5**	94±6	120±6	21±2	25±2	47±2	A
**Ori6**	94±6	104±6	19±2	26±2	45±2	A
			**CYP2C9:M3**			
**Ori1**	101±6	126±5	20±2	25±2	47±2	A/B
**Ori2**	92±7	116±6	18±2	24±2	46±2	A
**Ori3**	94±9	120±8	19±2	25±2	46±2	A
**Ori4**	92±7	139±7	22±2	24±2	47±2	B
**Ori5**	95±6	139±7	21±2	24±2	47±2	B
**Ori6**	95±6	138±6	22±2	24±2	48±2	B

* Mean and standard deviation for M1 Ori2 were calculated after 12 μs.

Table S3: CG simulations of mutant chimeras show the dependence on residues at the membrane interface of the final orientation of the globular domain with respect to the membrane. Values of the angles and distances, as well as the classes (A, A/B, B), characterizing the positioning of the globular domain are shown for CG trajectories of mt2C9 and mt2C19 started from 5 different initial conformations of the full protein with different orientations of the globular domain. Values of means and standard deviations were computed after excluding the first 8.5 microseconds for mt2C9 and the first (1) microsecond for mt2C19.

**mtCYPs Orientation***	**Angles (°)**		**Distances (Å)**			**Class**
	α	β	Linker	F'-G'	Globular	
			**mt2C9**			
**Ori1**	102±5	125±5	27±4	30±4	44±2	A/B
**Ori2**	99±6	139±6	28±4	29±4	45±2	B
**Ori3**	98±6	123±6	27±4	29±4	44±2	A
**Ori4**	93±7	133±10	27±4	29±4	44±2	B
**Ori5**	98±6	124±6	26±4	29±4	44±2	A
**Ori1-5**	98±7	129±10	27±4	29±4	44±2	A/B
			**mt2C19**			
**Ori1**	93±6	114±6	20±2	25±2	47±2	A
**Ori2**	94±8	139±8	18±2	24±2	47±2	B
**Ori3**	102±6	128±5	19±2	25±2	46±2	A/B
**Ori4**	94±8	116±12	18±2	24±4	45±2	A
**Ori5**	94±7	138±6	21±2	24±2	47±2	B
**Ori1-5**	95±8	127±13	19±2	25±3	46±2	A/B

*Initial orientations for all mutant simulations were the same as for the corresponding wildtype CG system (S1)

Supporting Figures:

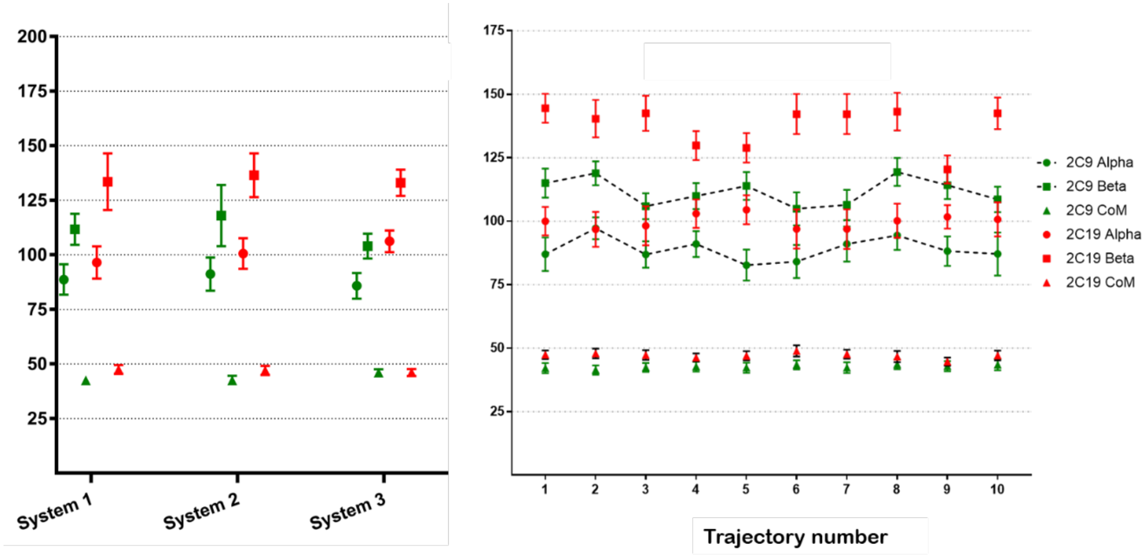

Figure S1: Comparison of the orientation and position of the globular domain with respect to the phospholipid bilayer in the CG simulations of wild-type CYP 2C9 (green) and CYP 2C19 (red). Angles α and β (°) and the axial distance of the CoM of the globular domain from the membrane CoM (Å) are given by circles, squares, and triangles, respectively. Mean and standard deviation values are given for systems S1-S3, defined in Table 1 (left) and for 10 trajectories of CG simulation systems 1 (S1) (right). The dotted lines connecting the CYP 2C9 angles are provided for guidance. Each trajectory was started with a different initial conformation of the flexible linker and therefore a different initial orientation of the globular domain. In the CYP 2C9 S1 simulations, all 10 trajectories gave β values below 125° (class A). In the CYP 2C19 S1 simulations, 8 trajectories gave β values above 130° (class B), 1 had a β value in the range 125° - 130° (class A/B), and one had a β value below 125° (class A). For all three systems (S1-S3), on average, CYP 2C9 adopted a class A orientation and CYP 2C19 adopted a class B orientation.

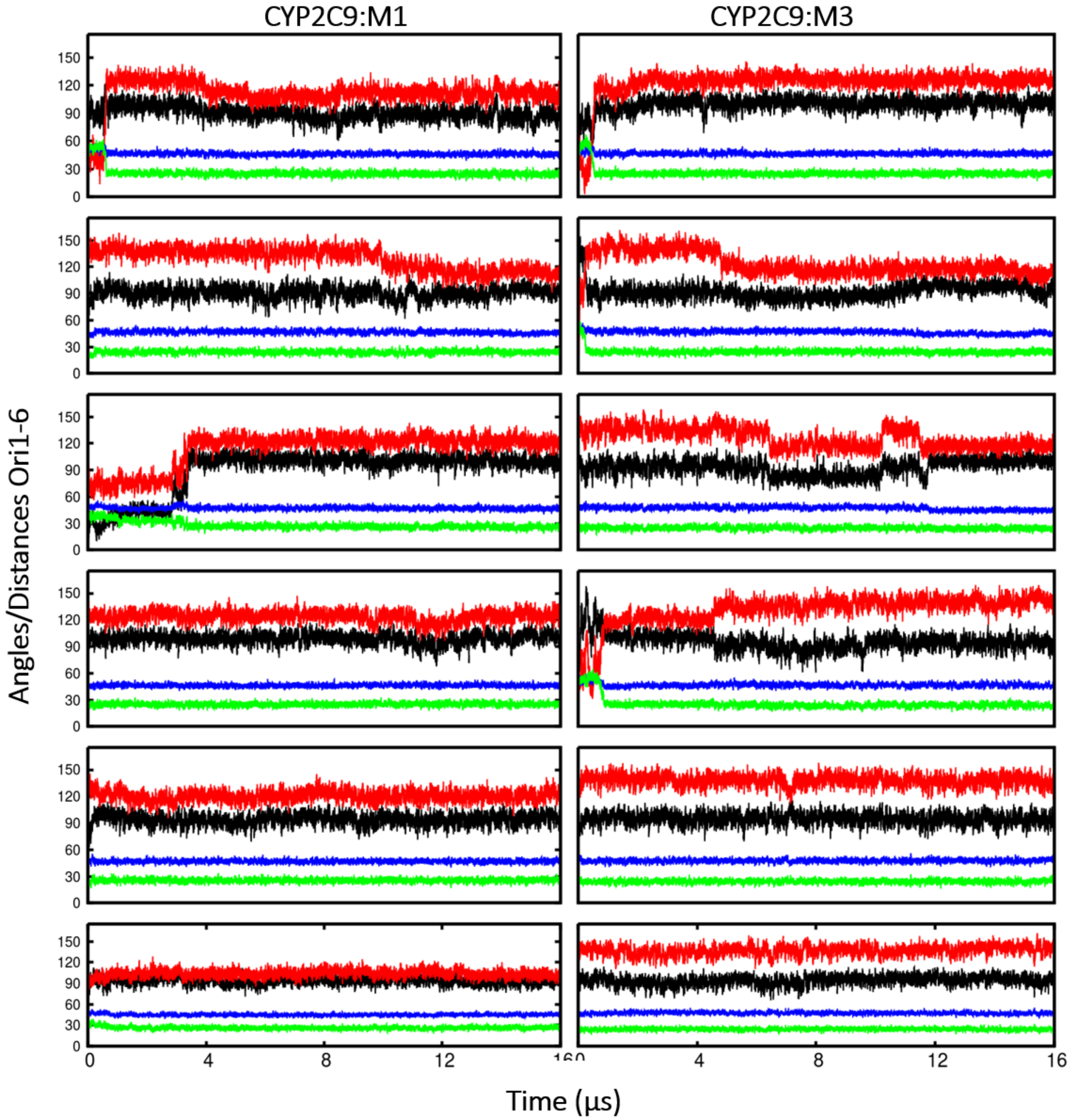

Figure S2: Convergence of the orientation and position of the globular domain during CG simulations of CYP 2C9 M1 (left) and M3 (right) systems. Angles (°) and distances (Å) are shown vs simulation time (μs) for six trajectories for each system. The color scheme is as in Figure 3.

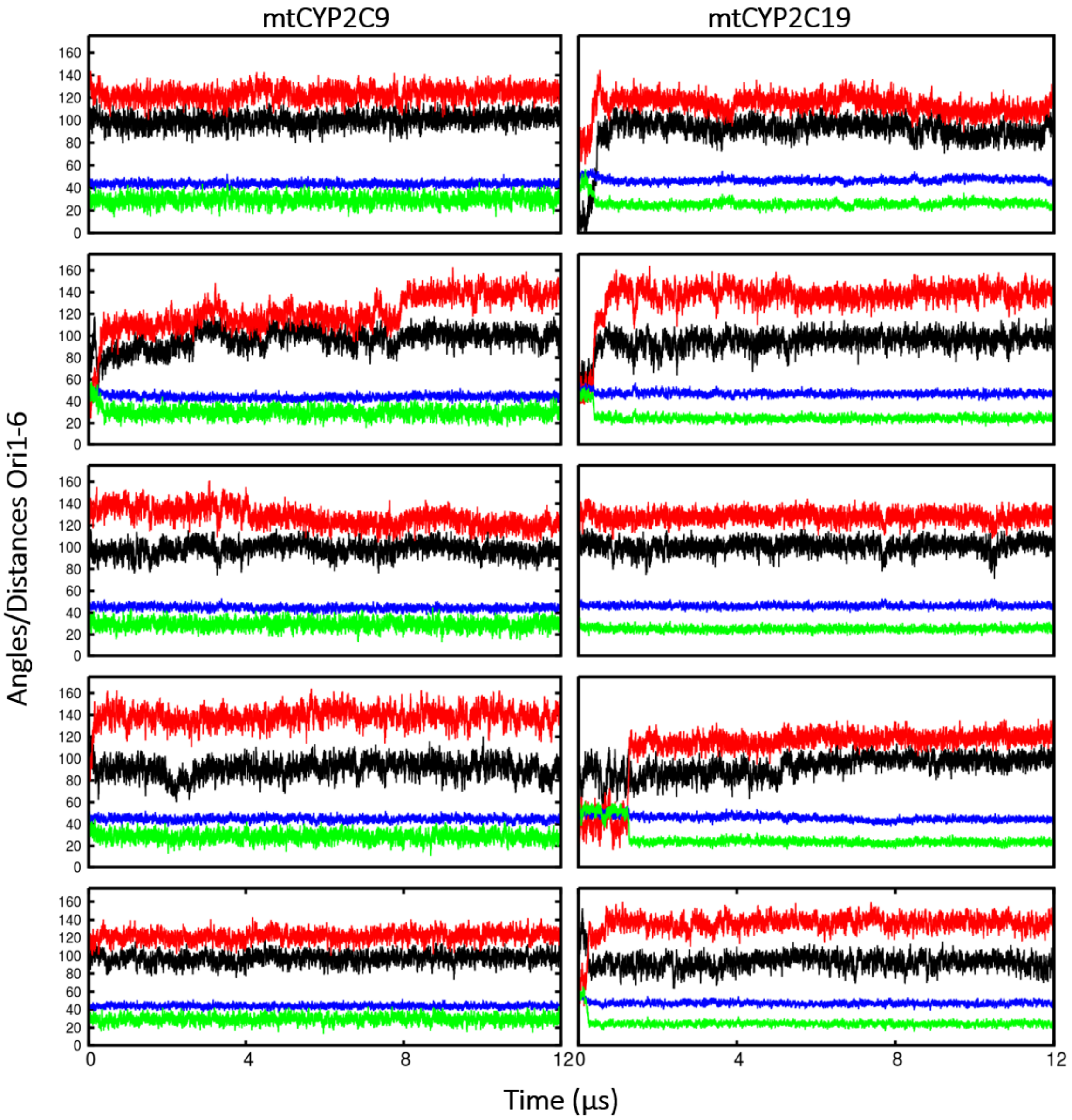

Figure S3: Convergence of the orientation and position of the globular domain during CG simulations of mt2C9 (left) and mt2C19 (right). Angles (°) and distances (Å) are shown vs simulation time (µs) for six trajectories for each system. The color scheme is as in Figure 3.

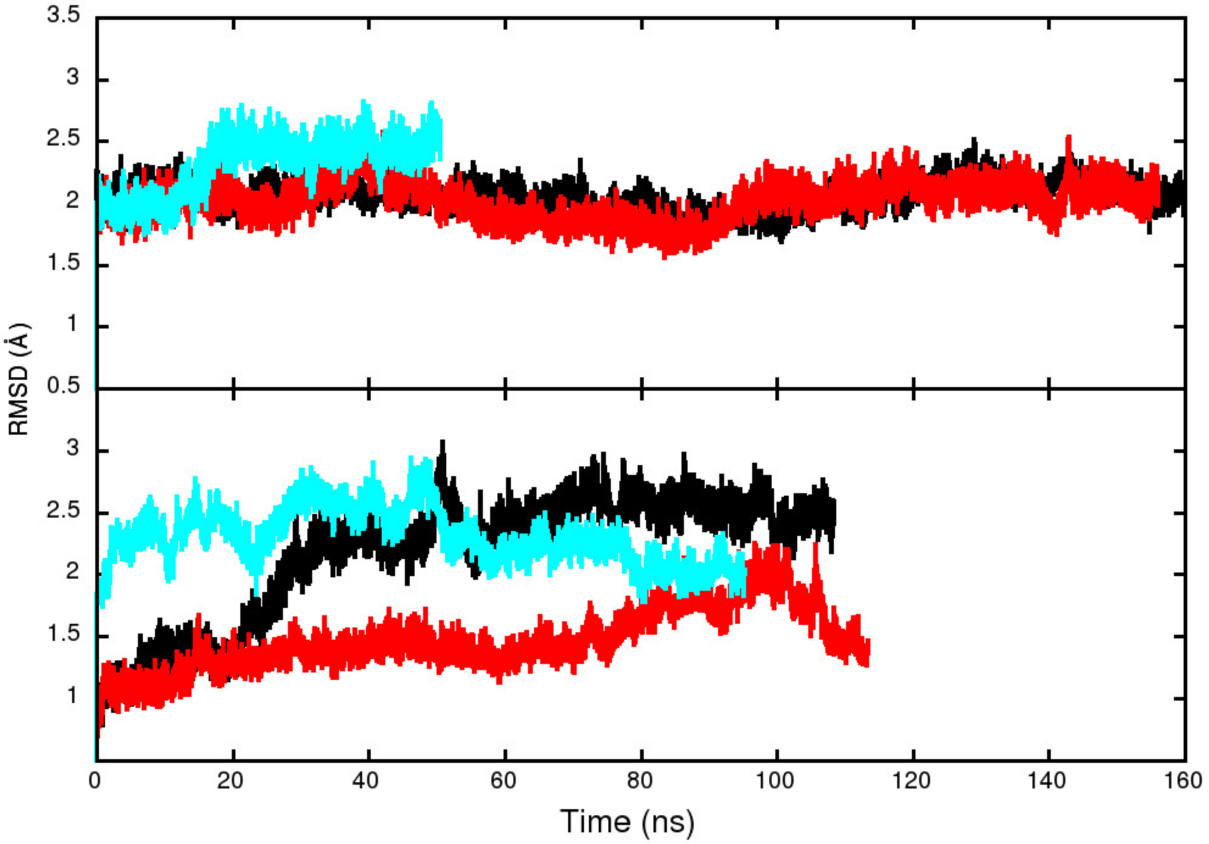

Figure S4: Cα-atom root mean squared deviation (RMSD) plots during AA MD simulations of the CYP2C9 (upper) and CYP2C19 (lower) globular domains (residues 50-490) with respect to the energy minimized structures are shown for simulations starting with the structure from CG simulation CG:S1 for apo (black) (SIM1) and ligand bound (red) (SIM2) forms and starting from CG:S2 for the apo form in cyan (SIM3).

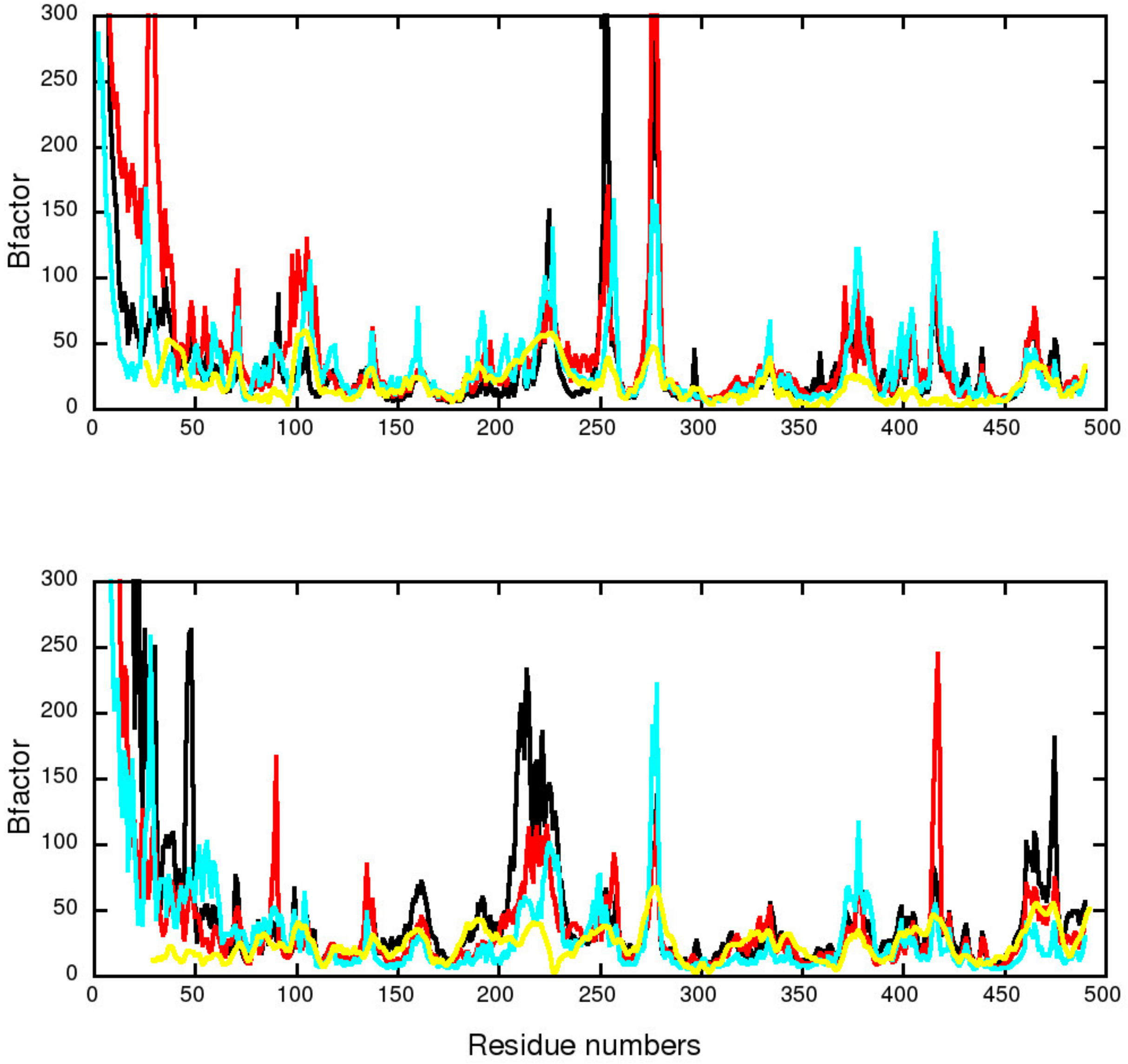

Figure S5: Comparison of the average B-factor values ($8\pi^2 rmsf^2/3$) of CYP 2C9 (top) and CYP 2C19 (bottom) in AA MD simulations with the crystallographic B-factors (yellow). Simulations are colored as in Figure S4.

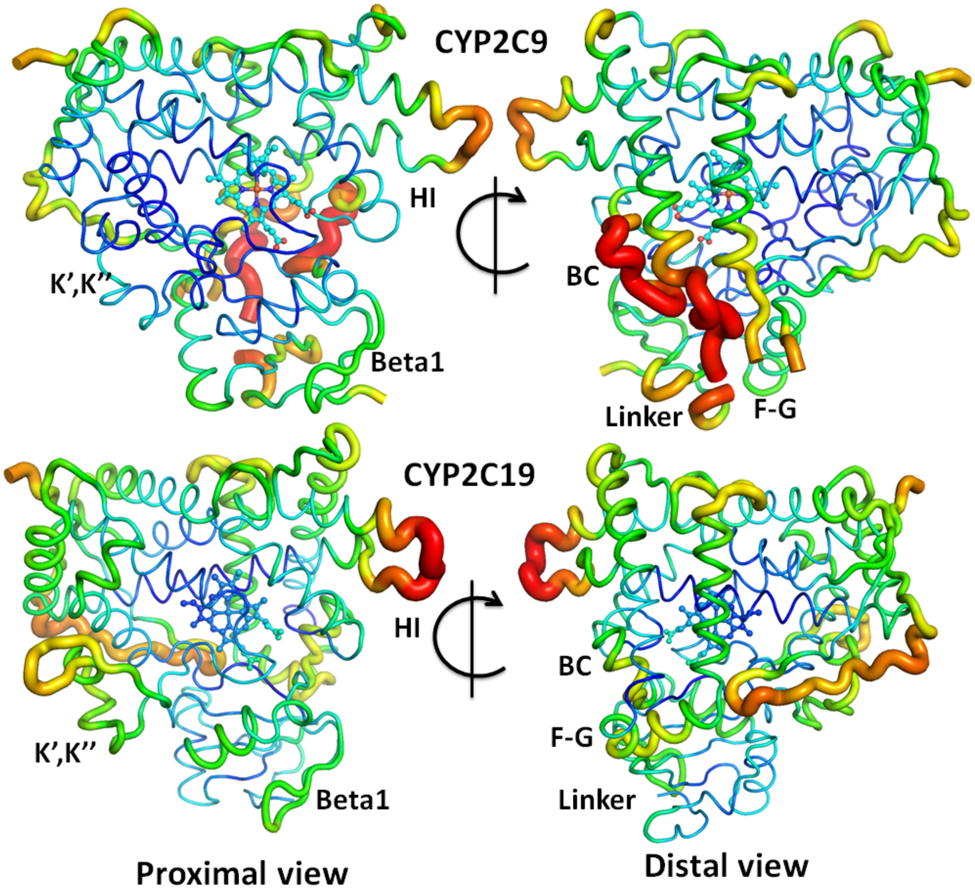

Figure S6: Crystal structures colored by B-factor values for the two CYPs (CYP 2C9: PDB 1R9O, and CYP 2C19: PDB 4GQS). The proximal view is from the side where the CYP reductase is expected to bind. The distal view is from the side where tunnels from the active site to the solvent open. Increasing B-factor is represented by color, from blue to red, and the thickness of the ribbon, from thin to thick. The heme is shown in stick representation.

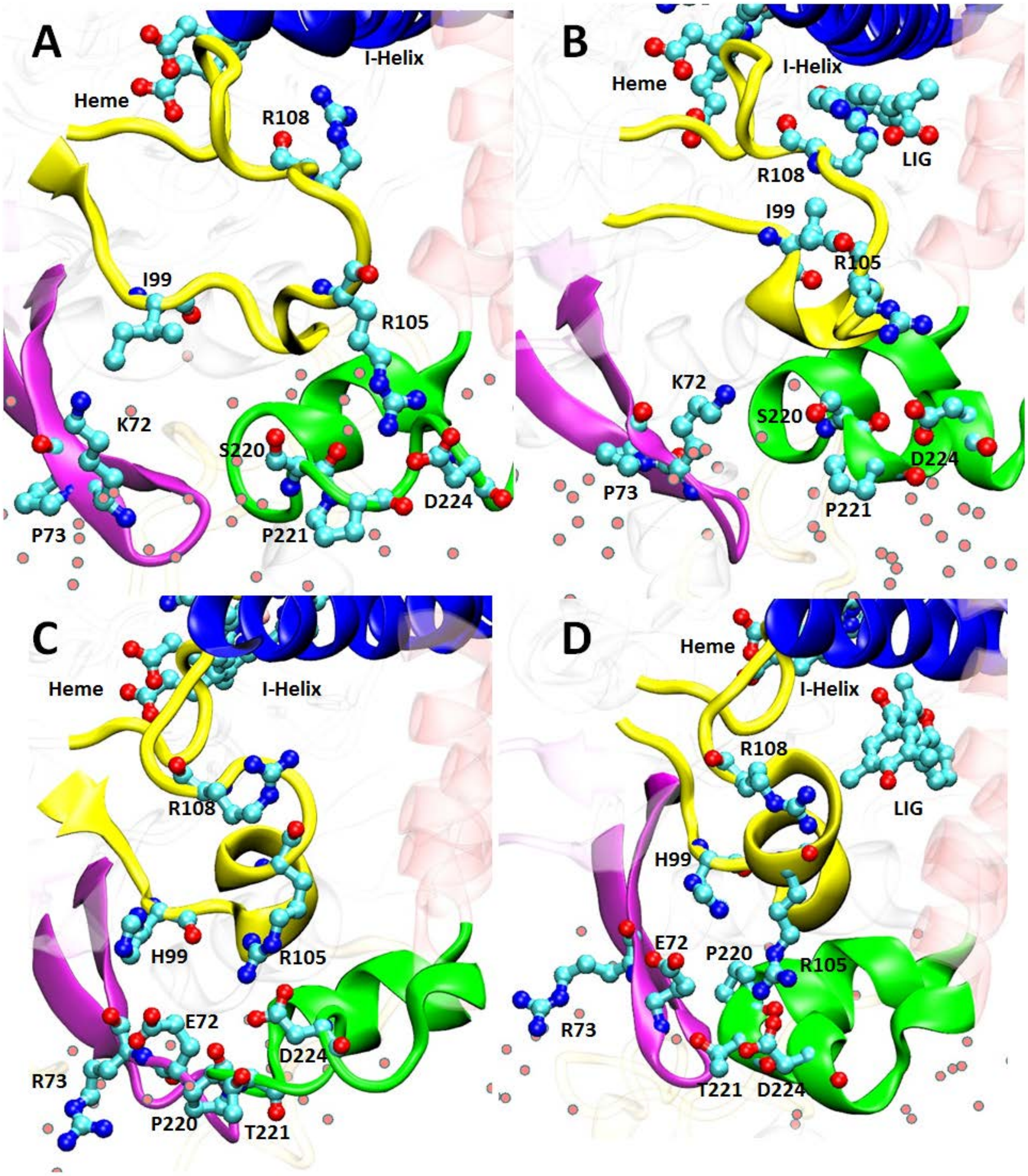

Figure S7: Differences in the CYP 2C9 and CYP 2C19 residues at the membrane interface shown in the last frames of AA MD simulations (SIM1 and SIM2). (A-D): The residues in CYP 2C9 apo form (A) and ligand (flurbiprofen) bound form (B) are shown in cyan colored stick representation. The protein is shown in cartoon representation with different colors assigned to secondary structures. β-sheet 1-2 is shown in violet, the BC loop in yellow, the F'G' helices in green, and the I-Helix in blue. The rest of the protein is shown in transparent grey and red color. The same representation is followed for the CYP 2C19 apo (C) and ligand (0XV) (D) bound forms.
